# Elucidation of cross-species proteomic effects in human and hominin bone proteome identification through a bioinformatics experiment

**DOI:** 10.1186/s12862-018-1141-1

**Published:** 2018-02-20

**Authors:** F. Welker

**Affiliations:** 1 Department of Human Evolution, Max-Planck-Institute for Evolutionary Anthropology, Leipzig, Germany; 2 Natural History Museum of Denmark, University of Copenhagen, Copenhagen, Denmark

**Keywords:** Palaeoproteomics, Error-tolerant proteomics, Bioinformatics experiment, Single amino acid polymorphisms, Hominidae

## Abstract

**Background:**

The study of ancient protein sequences is increasingly focused on the analysis of older samples, including those of ancient hominins. The analysis of such ancient proteomes thereby potentially suffers from “cross-species proteomic effects”: the loss of peptide and protein identifications at increased evolutionary distances due to a larger number of protein sequence differences between the database sequence and the analyzed organism. Error-tolerant proteomic search algorithms should theoretically overcome this problem at both the peptide and protein level; however, this has not been demonstrated. If error-tolerant searches do not overcome the cross-species proteomic issue then there might be inherent biases in the identified proteomes. Here, a bioinformatics experiment is performed to test this using a set of modern human bone proteomes and three independent searches against sequence databases at increasing evolutionary distances: the human (0 Ma), chimpanzee (6-8 Ma) and orangutan (16-17 Ma) reference proteomes, respectively.

**Results:**

Incorrectly suggested amino acid substitutions are absent when employing adequate filtering criteria for mutable Peptide Spectrum Matches (PSMs), but roughly half of the mutable PSMs were not recovered. As a result, peptide and protein identification rates are higher in error-tolerant mode compared to non-error-tolerant searches but did not recover protein identifications completely. Data indicates that peptide length and the number of mutations between the target and database sequences are the main factors influencing mutable PSM identification.

**Conclusions:**

The error-tolerant results suggest that the cross-species proteomics problem is not overcome at increasing evolutionary distances, even at the protein level. Peptide and protein loss has the potential to significantly impact divergence dating and proteome comparisons when using ancient samples as there is a bias towards the identification of conserved sequences and proteins. Effects are minimized between moderately divergent proteomes, as indicated by almost complete recovery of informative positions in the search against the chimpanzee proteome (≈90%, 6-8 Ma). This provides a bioinformatic background to future phylogenetic and proteomic analysis of ancient hominin proteomes, including the future description of novel hominin amino acid sequences, but also has negative implications for the study of fast-evolving proteins in hominins, non-hominin animals, and ancient bacterial proteins in evolutionary contexts.

**Electronic supplementary material:**

The online version of this article (10.1186/s12862-018-1141-1) contains supplementary material, which is available to authorized users.

## Background

The study of ancient protein sequences through tandem mass spectrometry (LC-MS/MS) represents a novel frontier in analyzing the phylogenetic placement of extinct species [1–3], including hominin populations [4], as well as the investigation of in vivo physiology, pathology, diet, and disease based on protein sequence analysis of archaeological and palaeontological samples [5–9]. Ancient proteins preserved in mineralized tissues provide phylogenetically informative amino acid sequences in fossils where no DNA survives [1], such as demonstrated by the recovery of collagen type I spectra from 3.4 Ma old Camelid bones in the Arctic [10] and 3.8 Ma eggshell proteins in central Africa [11]. This is in contrast to the oldest DNA sequences retrieved to date, which at approximately 0.7 Ma (from the permafrost) are significantly younger [12]. Ancient proteins therefore provide a biomolecular alternative in areas, time periods, and tissues where ancient DNA does not regularly survive [13].

Proteomics applied to mineralized tissues only provides insights into protein sequences from a subset of the genome - the largest ancient bone proteome published to date contains close to 200 proteins [14]. The total amount of phylogenetically informative positions is therefore drastically reduced in ancient proteomes compared to ancient DNA analysis of entire genomes, but such data can be retrieved from significantly larger periods of time. Hence, (hominin) fossils preserving no, little, or highly contaminated ancient DNA sequences might be amenable to ancient protein analysis. Previous research concluded that ancient protein sequences can be used to study hominin phylogeny, as 1) it is possible to retrieve hominin bone proteomes from Late Pleistocene fossils [4, 15], and 2) such bone proteomes contain single amino acid polymorphisms (SAPs, the protein analogue of single nucleotide polymorphisms, SNPs) known to differ between various clades of Late Pleistocene hominins for which ancient genomes are available (Neanderthals, Denisovans and modern humans; [4, 16, 17]). However, up to now there has been no demonstration that the bioinformatic analysis of such ancient hominin proteomes is also able to correctly infer novel SAPs when analyzing entire proteomes, as previously demonstrated for the protein collagen type I [1]. This needs to be demonstrated before moving on to the analysis of older and possibly more divergent hominin fossils.

The analysis of tandem mass spectrometry data in proteomics commonly either relies on the matching of MS/MS spectra to a protein sequence database of the target species by the use of a dedicated algorithm (for example MASCOT, Byonic or MaxQuant; [18–20]), or through de novo only protein sequencing without a provided protein sequence database (for example PEAKS de novo, NOVOR or PepNovo; [21–23]). De novo algorithms suffer from high rates of incorrect peptide sequence identifications, however [24]. Hence, the adoption of error-tolerant algorithms that utilize protein sequence databases while allowing sequence deviation (i.e., mutations) is currently the method-of-choice when identifying novel SAPs in palaeoproteomics. Several of the above-mentioned database matching algorithms now also provide error-tolerant options as add-on functionality, while others are designed specifically as error-tolerant search engines. PEAKS (Bioinformatics Solutions Inc.) in particular has recently been used in a number of studies investigating the phylogenetic potential of proteomes retrieved from now-extinct species or populations [1, 4, 25–29]. Such studies aim to utilize dedicated, restricted databases in order to keep estimated False Discovery Rates (FDR) low [30]. Hence, quite often a single reference proteome from the studied organism, or a closely related organism, will be included during bioinformatics analysis.

This presents a problem with the recovery of increasingly older protein datasets from species more distantly related to available (modern) reference proteomes [4]. With a larger number of sequence differences between the target sequence (present in the protein extract and in resulting MS/MS data) and the provided database sequence, more peptides and potentially proteins will remain unidentified in standard, non-error-tolerant searches as such searches do not allow mutations/substitutions between an identified sequence and the database sequence. Hence, issues associated with “cross-species proteomics” - the use of a protein sequence database from a species/population different from the target species/population – will become more prevalent in palaeoproteomics [31, 32]. Error-tolerant searches do allow for the identification of SAPs between an identified peptide sequence and the homologous database sequence. The ideal scenario would therefore be that error-tolerant searches entirely overcome the cross-species proteomics problem. Error-tolerant searches might instead only partly overcome the cross-species issue by identifying some mutation-containing spectra, but not all, introducing hitherto unknown biases in peptide sequence recovery and proteome composition. This could have adverse consequences for phylogenetic analyses conducted on ancient protein sequences from now-extinct organisms, including hominins, or the quantitative comparison of entire proteomes along diagenetic or evolutionary gradients.

Previous work has demonstrated that for collagen type I (COL1), PEAKS is capable of generating the correct amino acid sequence for modern and Pleistocene samples [1], as tested by obtaining proteomic sequence data of species for which the COL1 sequences were known but not included in the protein sequence database. On the proteome level, however, there is no demonstration of the effect that error-tolerant algorithms have on protein and peptide identification or its ability to correctly identify SAPs. It is therefore unknown to what extent error-tolerant searches overcome the cross-species problem in (palaeo)proteomics. Here, a bioinformatics experiment is performed to explicitly test non-error-tolerance and error-tolerance performance on a set of seven clinical, modern human bone proteomes available online [33].

**Fig. 1:**
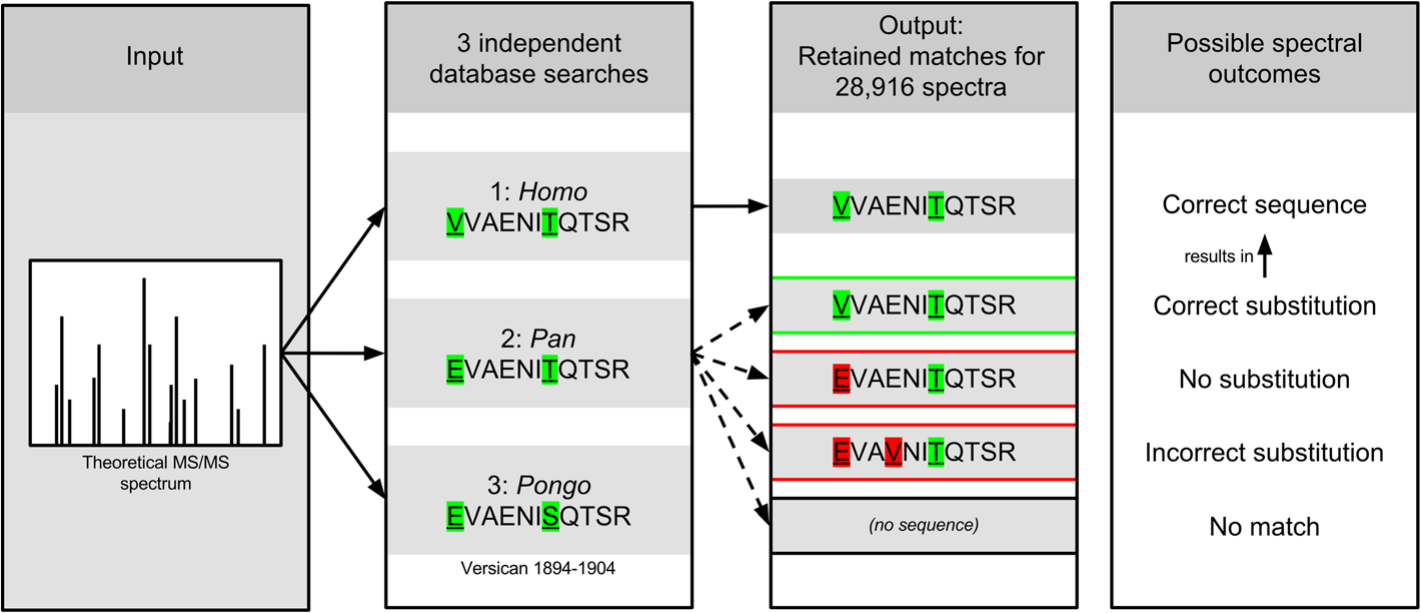
Experimental set-up and possible outcomes of an error-tolerant search for a mutable PSM. In the first search, MS/MS data is searched against the complete *Homo* database, resulting in ground-truth knowledge of 28,916 PSMs after necessary filtering. Subsequently, the same datasets are searched against the *Pan* and *Pongo* databases, respectively. The example concerns a mutable PSM with sequence differences between all three included databases. The only outcomes possible for a non-error-tolerant search or an identical, homologous PSM are the identification of the correct, non-mutated sequence (top) or no PSM match (bottom, outcome 4)

First, each bone proteome dataset was individually searched against the human reference proteome, thereby identifying the amino acid sequence represented by each PSM (peptide-spectrum match; Database 1). This search provides the ground-truth observation of protein and peptide composition. Next, the same spectral dataset is searched independently, but with similar search constraints, against database 2 (the *Pan* proteome) and subsequently against database 3 (the *Pongo* proteome; Fig. 1). The experiment thereby estimates protein identification, PSM identification and error-tolerance performance at evolutionary distances of approximately 0 Ma, 6-8 Ma, and 16-17 Ma, respectively [34–36]. Knowing the sequence differences between orthologous proteins for all three proteomes allowed establishing which of four possible outcomes occurred for each PSM individually in the searches against database 2 and 3: 1) the error-tolerant search identified the correct sequence, including a correctly placed amino acid substitution (SAP) of the right type, 2) no substitution suggested, 3) an incorrect substitution suggested, either at the wrong amino acid position or of the wrong type, or 4) the search to database 2 and 3 did not result in a PSM for the relevant spectrum. Outcome 1, the identification of the correct sequence, would represent a positive result, while outcomes 2 and 3 would represent a negative result with adverse consequences for subsequent phylogenetic analysis. In contrast, outcome 4 represents a negative result without further phylogenetic consequences, but with potential negative effects on protein identification. The performance of a standard, non-error-tolerant search is estimated simultaneously, as in such a search the only outcomes are spectral matches to non-mutated peptide sequences for identical, homologous peptides or the failure to identify phylogenetically informative, homologous sequences (outcome 4). This bioinformatic experiment therefore allowed establishing the effect of error-tolerant database matching on the total number of identified proteins, the number of PSMs matching to phylogenetically informative amino acid sequences, and the constraints on SAP identification for increasingly differentiated protein sequences.

## Methods

Publicly available data were taken from Salmon et al., [33], comprising three bone proteome datafiles for seven modern human individuals each. LC-MS/MS data (.mgf format) were searched in PEAKS v.7.5 [22] against the reference proteomes from *Homo sapiens*, *Pan troglodytes* and *Pongo abelii*, respectively (hereafter referred to as the *Homo*, *Pan* and *Pongo* databases). Reference proteomes were downloaded from UniProt on 30/06/2016 using the canonical sequence for each protein only. PEAKS searches included the full set of available processes (PEAKS de novo > PEAKS DB > PEAKS PTM > PEAKS SPIDER). Within this workflow, PEAKS SPIDER is restricted to the identification of one amino acid mutation compared to the reference database, while PEAKS DB and PEAKS PTM do not have this constraint (Dan Maloney, pers. comm. 2017). The workflow also allows the identification of semitryptic and non-tryptic peptides, which are a common feature of ancient proteome datasets [4, 8].

A protein list containing commonly identified contaminants, composed of proteins listed on the common Repository of Adventitious Proteins and those mentioned in reference [37], was added to each reference proteome (see Additional file 1). No further modifications were made to the databases downloaded from UniProt to accurately mimic the strategy that researchers commonly follow when constructing reference databases using a single proteome from a target or closely related species. Search settings were identical to those reported in Salmon et al., [33], with carbamidomethylation as a fixed post-translational modification, methionine oxidation, phosphorylation of serine, threonine and tyrosine, glutamine and asparagine deamidation, and proline hydroxylation as variable modifications, in line with the extraction method used by Salmon et al., [33], the proteome studied (dominated by hydroxylated collagen), and diagenetically induced protein modifications (glutamine and asparagine deamidation). One missed cleavage was allowed, with a precursor error tolerance of 10 ppm and 1 Da for fragment ions. Proteins identifications were accepted when at least two unique peptide-spectrum matches (PSMs) were present in the first search (against the *Homo* database), and PSMs were only accepted with a False Discovery Rate (FDR) equal to 1.0%.

For evaluation of error-tolerant performance, PSMs were only retained if that particular scan had been matched in the search against the *Homo* database and when the PSM had a length of ≥10 amino acids. Furthermore, all PSMs that matched to proteins that are either absent, or have sequence regions wrongly predicted, in the *Pan* or *Pongo* database were removed from the dataset entirely or partly, respectively. This included protein sequences translated from the following genes: *COL6α1*, *COL3α1*, *COL1α1*, *COL5α1*, *IGHG1*, *IGHG2*, *COL22α1*, *GLIS1*, *VIME*, *H2B2E*, *ITIH1*, *ANXA6*, *COL19α1*, *IGKC*, *TGFBI*, *CLEC3B*, *SLC25α6*, *HSPG2*. Finally, PSMs matching to proteins commonly suspected to represent contaminants in bone proteomics were also removed (including *ALB*, *HBB*, *HBA*, *IGF2*, *GELS*, *ANXA5*, *RS27A*, *K2C1*, *K1C16*, *K1C10*, *K1C9*). Despite these rigorous filters, a total of 28,916 PSMs (65.2% of the total PSMs initially present) were retained. The majority of removed PSMs was due to sequence length (< 10 amino acids, 29.1% of the total PSMs initially present).

For each PSM, the evolutionary distance between the amino acid sequences retrieved in the search against the *Homo* database and its orthologous sequence present in the *Pan* and *Pongo* databases can be quantified as the number of amino acid differences (SAPs). PSMs with an evolutionary distance of 1 or greater, e.g., where an amino acid sequence difference exists between the used database and the target data (human), are termed “mutable PSMs”. Mutable PSMs can either be observed (outcomes 1, 2 or 3) or unobserved (outcome 4) during searches against the *Pan* and *Pongo* databases, and their sequences can be inferred correctly (outcome 1) or incorrectly (outcomes 2 and 3) in error-tolerant searches.

Mutable PSMs differing between orthologous sequences by leucine (L) <> isoleucine (I) substitutions were categorized as correctly identified as mass spectrometry commonly cannot differentiate between these two isobaric amino acids. Furthermore, isobaric post-translational modifications of amino acids can be misidentified as amino acid modifications – or vice versa. Here, taking into account the fragment ion tolerance of 1 Da, these include the detection of lysine formylation (equal in mass to a lysine (K) to arginine (R) substitution), and glutamine (Q) and asparagine (N) deamidation (equal in mass to substitutions towards glutamic acid (E) and aspartic acid (D)). They were not regarded as wrongly identified mutable PSMs because of the isobaric nature of their modifications.

To further investigate biases in the identified proteomes in the second and third search, d_N_/d_S_ values were computed to investigate protein evolution and selection acting on the identified proteins. d_N_/d_S_ represents the ratio of synonymous to non-synonymous (d_N_/d_S_) nucleotide substitutions and were obtained for each protein identified in the *Homo* search. Values were retrieved from ENSEMBL for protein orthologous with both *Pan* and *Pongo* using an in-house R script utilizing biomaRt (v. 2.22.0; [38]). High d_N_/d_S_ values of 1 or above generally indicate positive Darwinian selection, while low values close to 0 indicate purifying selection. The vast majority of proteins display values close to 0, reflective of the conserved nature of protein amino acid sequences in general.

Phylogenetic trees were constructed using amino acid alignments consisting of phylogenetically informative amino acid positions that were identified in the sample dataset and orthologous positions in the *Pongo abelii*, *Pan troglodytes* and *Homo sapiens* reference proteomes. Analysis of these alignments was conducted using PhyML in Geneious and RAxML on the CIPRES Science Gateway [39]. RAxML was run for 1000 bootstrap iterations using the Dayhoff substitution model (selected after running PartitionFinderProtein; [40]). PhyML was run using 10,000 bootstrap iterations. *Pongo* was specified as outgroup in both RAxML and PhyML phylogenetic analysis.

## Results

Proteome size of the seven studied individuals ranges between 31 and 54 proteins. This proteome size is similar to bone proteomes observed in some modern and some ancient bone proteome studies [4, 9, 41, 42]. The identified proteomes contain common non-collagenous bone proteins such as osteomodulin (*OMD*), alpha-2-HS-glycoprotein (*AHSG*), chondroadherin (*CHAD*), osteocalcin (*BGLAP*), and biglycan (*BGN*), as well as several collagenous proteins (primarily peptide-spectrum matches to collagen type I alpha 1 (*COL1α1*) and alpha 2 (*COL1α2*)). Collagen type 10 alpha-1 (*COL10α1*) is absent from the dataset, but this is not surprising as the studied bone samples derive from adult humans [33] while *COL10α1* is excreted by hypertrophic chondrocytes during initial bone mineralization and should disappear quickly from the bone proteome during bone remodeling later in life [4]. Nevertheless, several proteins that are identified here are known to contain phylogenetically informative SAPs for Late Pleistocene hominin clades for which (ancient) genomes are available (modern humans, Neanderthals and Denisovans [4, 16, 17]), while others are phylogenetically informative within Hominidae in general (see below). Both the size and composition of the studied proteomes is therefore comparable to proteomes retrieved from Late Pleistocene and Holocene bone specimens containing no or low amounts of endogenous DNA sequences [4, 42]. Some of these proteins are known to survive for particularly long periods of time, as shown by their recovery from Early Pleistocene sites in Europe [41].

### Error-tolerance performance

**Fig. 2:**
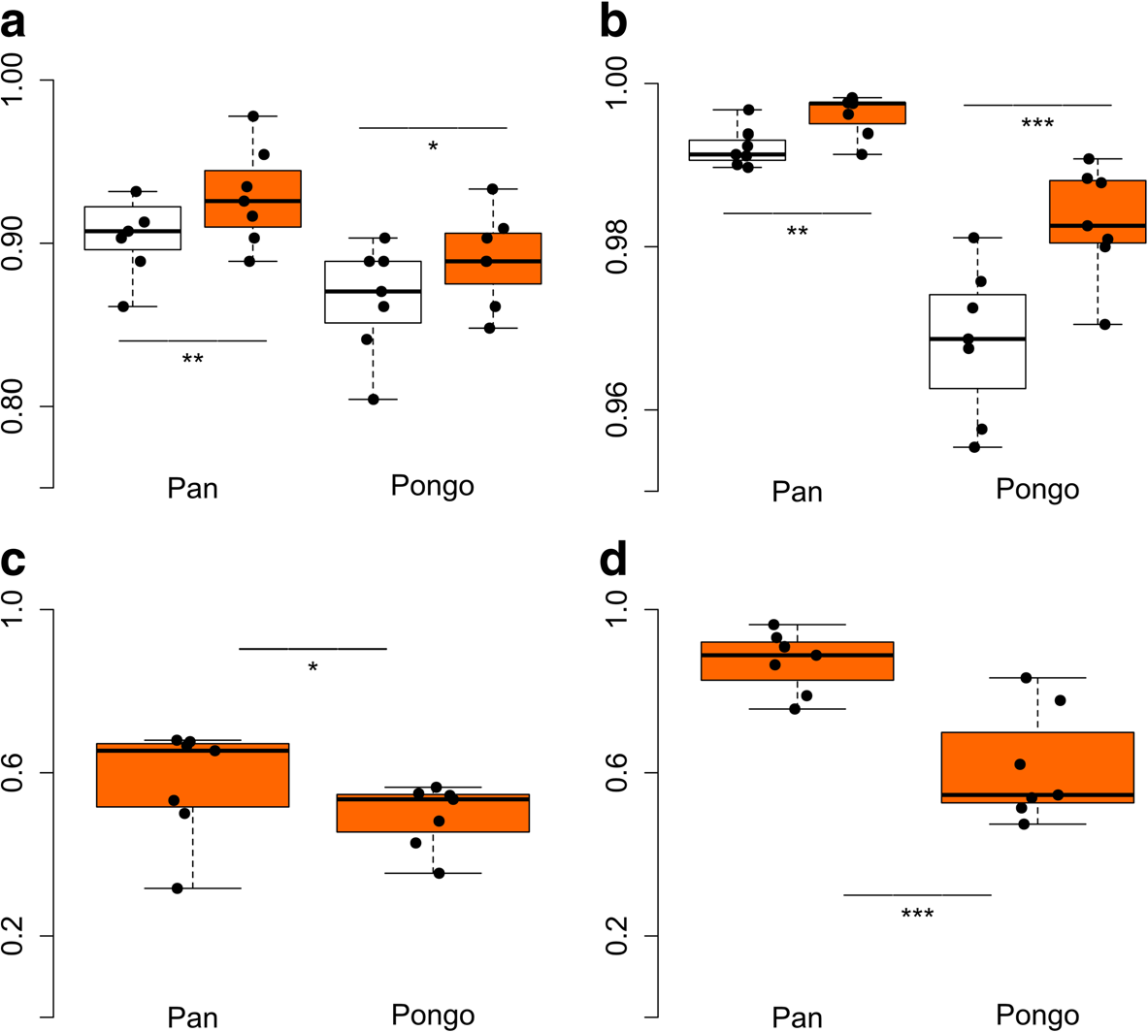
Performance of error-tolerant searches compared to non-error-tolerant matches in the same search. **a** Number of matched proteins. **b** Total number of Peptide Spectrum Matches (PSMs). **c** Number of observed mutable PSMs. **d** Retrieval of mutable positions in searches 2 and 3, regardless of the number of matching mutable PSMs. For **a** and **b**, open boxplots indicate a search equaling a non-error-tolerant search and a filled boxplot indicates an error-tolerant search. Values are normalized to the *Homo* database search per dataset. Note differences in y-axes. Statistical differences were determined by paired samples *t*-tests (* = *p* < 0.05, ** = *p* < 0.01, *** = *p* < 0.001)

A reduction in the number of identified proteins and peptides for error-tolerant and non-error-tolerant matches in searches against both the *Pan* and *Pongo* database is apparent (Fig. 2). A non-error-tolerant search would have resulted in, on average, 8.5% less protein identifications and 0.8% less PSMs in *Pan* and 13.1% less protein identifications and 3.2% less PSMs in *Pongo*. The error-tolerant search partly recovered data loss but not completely. Here, on average, 7.1% less protein identifications and 0.4% less PSMs in *Pan* and 11.0% less protein identifications and 1.7% less PSMs in *Pongo*. These differences between non-error-tolerant and error-tolerant searches are significant for both databases (Fig. 2). The reduction in proteome size is not correlated with the initial size of the proteome in the *Homo* search (r(13) = 0.24, *p* = 0.41). The reduction in both the number of identified proteins and PSMs is driven by a smaller number of observed mutable PSMs (Fig. 2c; as determined in the search against the *Homo* database), of which, on average, 47.5 and 49.3% were identified in the *Pan* and *Pongo* database search, respectively. This significant reduction in the number of observed mutable PSMs is not entirely reflected in whether *any* mutable PSMs were retained for each amino acid position of phylogenetic interest (Fig. 2d). Here, on average 87.2% positions of phylogenetic interest were recovered in the search against the *Pan* proteome, while on average only 61.4% of such positions were observed in the *Pongo* proteome. Hence, error-tolerant searches only partly overcome the problem posed by cross-species proteomics. They are less effective at larger evolutionary distances not only at the PSM level, where they would be expected, but especially at the protein level.

### Mutable and mutated PSM behaviour

**Fig. 3:**
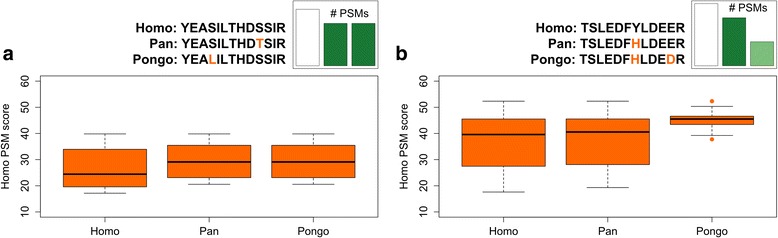
High-scoring PSMs are more likely to be identified in error-tolerant searches. **a** When *Pan* and *Pongo* both contain one SAP, but at a different sequence location, the same set of high-scoring PSMs is identified in the error-tolerant searches. **b** PSMs with lower scores are unidentified at increased evolutionary distances. Protein and peptide locations are Fibrinogen gamma chain 122-134 (*FGG*; FIBG_HUMAN) and Pigment epithelium-derived factor 226-237 (*SERPINF1*; PEDF_HUMAN), respectively

**Fig. 4:**
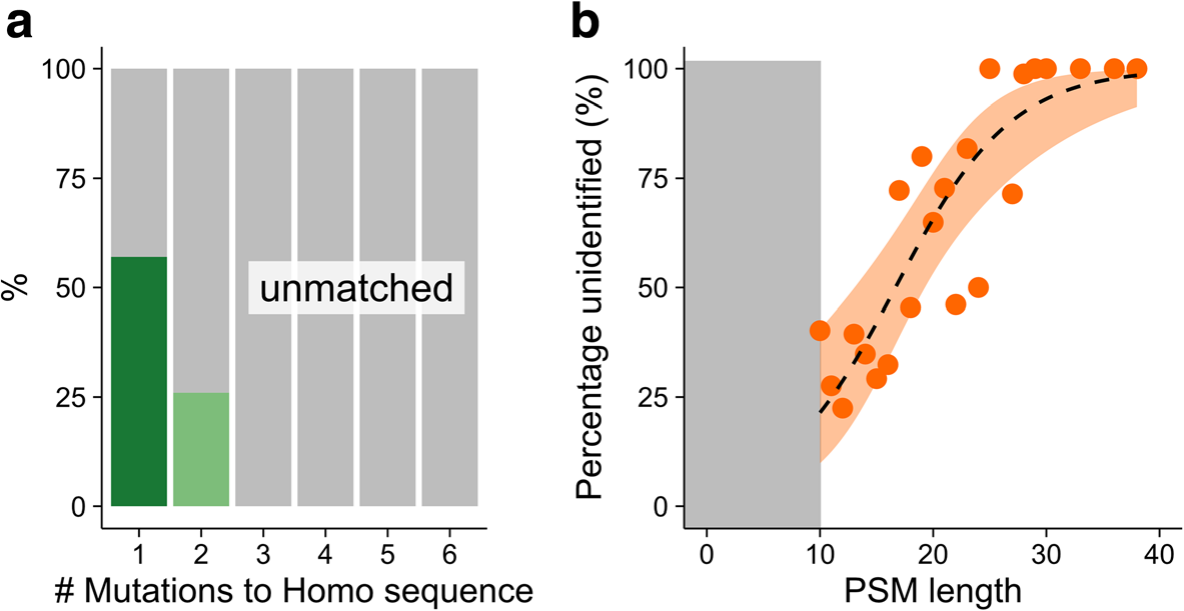
Characteristics of identified and unidentified mutable PSMs. **a** PSMs separated by evolutionary distance, here indicated as the number of amino acid differences (SAPs) between the homologous *Homo* and *Pan* or *Pongo* peptide sequence. Mutable PSMs are mostly identified when one amino acid position differs between the sequence represented by the MS/MS spectrum and the provided sequence database. **b** Proportion of unidentified mutable PSMs by PSM length. Longer mutable PSMs are frequently unidentified in the *Pan* and *Pongo* searches. Fitted logistic curve with 1SD. PSMs with a length of < 10 amino acids were excluded from analysis

The experiment allows evaluating the occurrence of the four possible outcomes of an error-tolerant search conducted on mutable PSMs. Either the correct substitution is identified (outcome 1), no substitution is identified (outcome 2), a substitution of the wrong type or position is identified (outcome 3), or there is no PSM match (outcome 4). As noted above, roughly half of the mutable PSMs were not recovered in either the *Pan* or *Pongo* database search (Fig. 2c), making outcomes 1 and 4 roughly equally prevalent. The unobserved mutable PSMs (outcome 4) are not randomly distributed within the datasets available, however. First, mutable PSMs that have a low peptide score in the *Homo* search are less likely to be matched in subsequent searches, regardless of the mutable position within the amino acid sequence (Fig. 3a). This trend is more extreme at larger evolutionary distances (Fig. 3b), and in agreement with general proteome results (Fig. 2). This observation is not surprising as higher-scoring PSMs tend to have more high intensity, low error internal fragment ions (MS2). Second, the evolutionary distance between homologous peptides has a significant effect on the ability of PEAKS to correctly identify SAPs. Just over half (57%) of the PSMs with one mutable position were recovered, roughly a quarter with two mutable positions (26%), and no PSM with an evolutionary distance of three or more amino acids was identified in subsequent searches to the *Pan* or *Pongo* database (Fig. 4a). Third, mutable PSMs with an amino acid length of 25 amino acids or more were almost never identified, while mutable PSMs with an amino acid length of between 10 and 15 amino acids were identified in up to 75% of cases (Fig. 4b). This holds true regardless of the evolutionary distance between the target and database sequence. No influence of changes in peptide mass, peptide charge, or peptide isoelectric point were observed (Additional file 2). It is difficult to test a relationship between peptide length and peptide score given the filtering criteria imposed on length (PSM length ≥ 10) and minimum score (FDR rate equals 1.0%), and a generally lower number of longer PSMs. Nevertheless, there seems to be an absence of such a correlation (Pearson correlation coefficient = − 0.09). To summarize, the evolutionary distance between the target sequence and the database sequence and PSM length both play a significant role in determining if a mutable PSM is identified.

No mutable PSMs were identified for which a mutation should have been suggested in the searches against the *Pan* and *Pongo* databases but where this did not happen (i.e., outcome 2). Theoretically, outcome 2 could occur by positioning a post-translational modification (PTM) equal in mass to the needed amino acid substitution on or nearby a mutable PSM in the searches against the *Pan* or *Pongo* database. Such PTMs could be non-specified PTMs as well, as PEAKS SPIDER has the ability to match PSMs containing such non-specified PTMs, theoretically causing a situation where (non-specified) PTMs and amino acid substitutions with the same mass modification are equally likely outcomes. Instead, such mutable PSMs seem to be lost from the dataset entirely. This implies that the possible confounding influence of outcome 2 is negligible in palaeoproteomic studies.

Estimating the influence of outcome 3, the identification of an amino acid substitution (SAP) at the wrong place or of the wrong kind, is more difficult to quantify. Error-tolerant searches have the tendency to also provide spurious PSM matches with incorrectly suggested mutations to PSMs that should not be mutated. These “off-target” suggested mutations have the potential to confound subsequent phylogenetic analysis as in normal situations one cannot differentiate a priori between off-target mutations and outcome 3 for mutable PSMs. Instead, here all mutated PSMs are quantified as correctly or incorrectly identified based on the a priori knowledge provided by search 1. Subsequently, mutation-containing PSMs were accepted or rejected based on 1) the presence of at least two PSMs covering the mutated amino acid position, and 2) these two or more PSMs comprise the majority of the total number of PSMs matching that amino acid position. Such filtering criteria mirror those utilized with PEAKS elsewhere [1, 4, 25]. Prior to filtering, roughly 20% of proposed mutations are incorrect (Table 1). However, after filtering 100% of mutation-containing PSMs derived from the searches against both the *Pan* and *Pongo* databases are correct. As a result, error-tolerant searches are correct when simple filtering criteria are implemented. The most important criterion seems to be to only accept novel SAPS with the support of multiple PSMs.

**Table 1:** Validity of suggested amino acid mutations (SAPs)

	Unfiltered		Filtered	
	*Pan*	*Pongo*	*Pan*	*Pongo*
1 mutation (incorrect)	18.8% (32)	15.2% (66)	–	–
1 mutation (correct)	81.2% (138)	80.9% (352)	100% (125)	95.6% (328)
2 mutations (incorrect)	–	–	–	–
2 mutations (correct)	–	3.9% (17)	–	4.4% (15)
Total PSMs	170	435	125	343

Filtering is based on a majority of matching PSMs containing the suggested mutation for a particular position *with* a minimum of two mutated PSMs

## Discussion

### Error-tolerant search outcomes

The bioinformatics experiment performed here allows estimating the occurrence of the four possible error-tolerant outcomes for mutable PSMs, thereby providing insights into which parameters influence error-tolerance performance at the peptide level. Simultaneously, the experiment allows comparison of standard, non-error-tolerant searches and error-tolerant searches at the protein and proteome level. The experiment was set up in such a way to include the complete PEAKS workflow (from de novo to SPIDER), without any further interference in provided reference proteome databases.

Correctly suggested substitutions account for roughly 80% of all suggested substitutions (Table 1), but simple filtering increased this to 100%. This provides experimental support for the use of error-tolerant search algorithms in ancient (hominin) protein analysis. Mutable PSMs were only identified when the evolutionary distance between the *Homo* and *Pan*/*Pongo* sequence was either 1 or 2 amino acids. PSMs with larger evolutionary distances were never identified in the error-tolerant searches. Furthermore, PSMs “identifiability” is severely impacted by PSM length, with long mutable PSMs almost never identified. Therefore, unidentified mutable PSMs are not randomly distributed in the dataset, or solely related to MS/MS quality. The disappearance of PSMs with longer amino acid sequence lengths as well as the absence of PSMs with three or more substitutions can either be explained by an unacceptable increase in search space within error-tolerant mode or the requirement of an unlikely number of high intensity internal fragment ions (or a combination of both). Furthermore, the number of consecutively database-sequence matching fragment ions and peptide length are utilized by PEAKS as two of the attributes involved in normalizing PSM scores, and so interruption of this database-matching series due to the presence of a SAP negatively impacts the identification of SAPs in longer PSMs in particular [43]. Although one might aim to optimize protein sequence coverage by generating longer peptide sequences through the use of different enzymatic digestion approaches, the experiment conducted here strongly indicates that this is a disadvantageous approach in error-tolerant experiments. Palaeoproteomic experiments aiming to provide molecular data on the phylogenetic placement of extinct populations or species, be they human, hominin, or animal, should instead focus their efforts on generating large amounts of relatively short peptide sequences. This can be achieved through changes in extraction chemistry and/or mass spectrometry set-up.

Incorrect substitutions seem to account for roughly a fifth to a quarter of the suggested amino acid mutations when left unfiltered. Previously, in the context of collagen type I phylogenetics, it has been suggested to only accept suggested amino acid substitution when these were present in two independent MS/MS spectra and these mutation-containing spectra formed the majority of all PSMs matching to that particular amino acid position [1]. In that study and elsewhere, such substitutions are subsequently verified by re-searching the entire spectral dataset against a new sequence database containing the modified protein sequences. Here, such re-searching was not performed but it is shown that similar filtering criteria remove all falsely suggested amino acid substitutions (Table 1). This comes at a cost, as correctly substituted PSMs that are the only matching PSM to a particular phylogenetically informative position are also filtered out. Running the same protein extract several times on a tandem mass-spectrometer would be a simple way of overcoming this issue, simultaneously demonstrating replicability of generated tandem mass spectrometry results overall.

### Proteome composition and retrieval

Recent comparisons of bone proteomes have provided vital insights into proteome degradation [41] and changes in bone proteomes composition during maturation and aging [14]. Such studies rely on the comparison of proteomic datasets derived from model organisms that are searched against available reference proteomes. Hence, and based on the results of this study, they do not suffer from issues arising from cross-species proteomic approaches. The inclusion of non-model or extinct organisms into such comparisons would be more complicated at moderate or larger distances such as the *Homo*-*Pan* split (approximately 6-8 Ma). As the data indicates here, protein identification loss can be 10% or more, even when using an error-tolerant search (Fig. 2b). As ancient proteome datasets are (sometimes) relatively small, the missed identification of a particular protein can have a proportionally large influence on proteome interpretation.

**Fig. 5:**
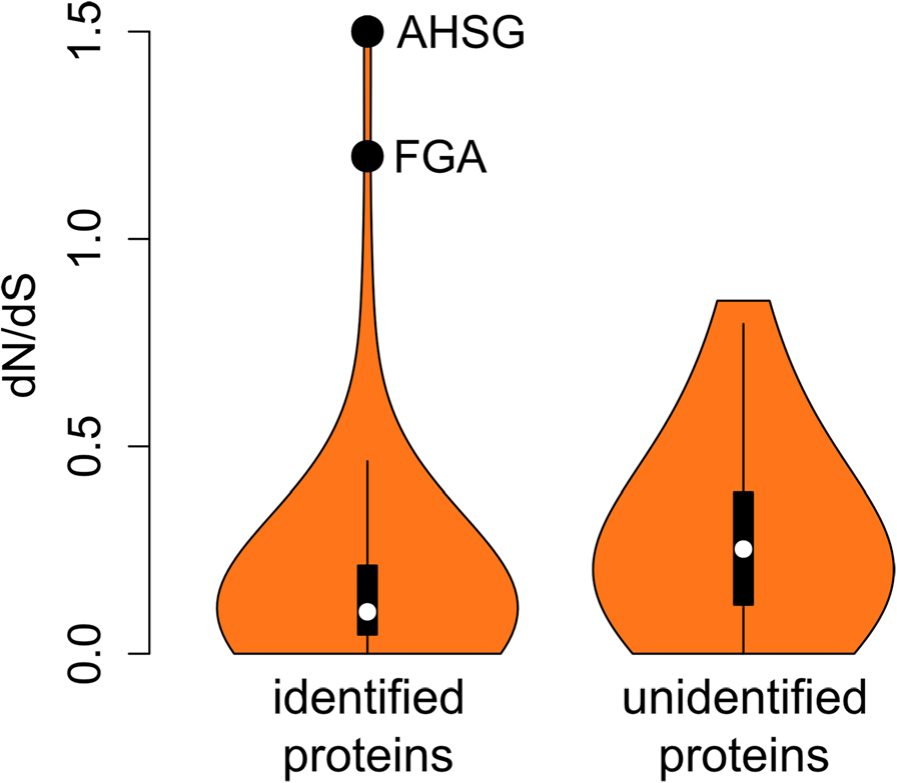
Violin plot of the ratio of non-synonymous to synonymous (d_N_/d_S_) scores for proteins identified and unidentified in searches two and three in error-tolerant mode. Protein identifications are compared to the search against the human reference proteome (search one). d_N_/d_S_ values between the identified proteins and the unidentified proteins is moderately different (t.test(− 2.65), df = 56.131, *p* = 0.01)

Fast evolving proteins observed in ancient protein datasets are of particular interest as they provide increased phylogenetic information compared to slow-evolving proteins. Based on the results presented here, such proteins might instead remain unidentified in error-tolerant searches exactly because of their high substitution rates. Fast-evolving proteins in ancient protein studies are not restricted to bone proteins in evolutionary applications but also include (human) proteins involved in immune response, bacterial proteins recovered from dental calculus (due to higher mutation rates and horizontal gene transfer), and potentially proteins under positive or negative selection during domestication processes [7, 8, 44]. Computation of d_N_/d_S_ values of orthologous proteins (*Homo*-*Pan* and *Homo*-*Pongo*) provides some insights into the distribution of fast-evolving, potentially positively selected proteins among the different proteomes obtained during the three searches. Comparison of d_N_/d_S_ values for retained proteins and missed proteins indicates that there is indeed a moderately significant difference in proteome composition, even with error-tolerant searches (Fig. 5). These observations are consistent with a previous non-error-tolerant search in a cross-species analysis of mammalian sperm proteins [31], suggesting that fast-evolving, potentially positively selected proteins are prone to remain unidentified in both standard and error-tolerant proteomic searches in a wide range of possible ancient protein studies.

It is of note that two proteins, alpha-2-HS-glycoprotein (*AHSG*) and fibrinogen alpha chain (*FGA*), with d_N_/d_S_ > 1 are still identified in searches 2 and 3. This suggests that the phylogenetically informative positions of these two proteins are sufficiently spaced apart to ensure that error-tolerant searches are capable of identifying novel amino acid sequences even at large evolutionary distances (at the protein level). These two proteins are also frequently observed in ancient bone proteome datasets, making them prime targets for future phylogenetic studies [12, 37, 41, 45]. Furthermore, the presence of proteins in the bone proteome that are potentially under positive selection in hominin evolution requires further study.

### Phylogenetic implications

The use of ancient protein datasets to reconstruct the phylogenetic position and evolutionary history of extinct species relies on the identification of amino acid sequence differences (SAPs) between orthologous proteins. In the past years, this approach has largely focused on the use of error-tolerant search algorithms of tandem mass spectrometry datasets against a closely related reference proteome, particularly the PEAKS workflow. The identification of proteins with large amounts of amino acid sequence differences between orthologous proteins in ancient proteomes has been seen as particularly significant for such purposes [41], especially in combination with other less variable proteins such as collagen type I that are more consistently identified and potentially observable for longer periods of time in the geological record [1–4, 25, 26].

**Fig. 6:**
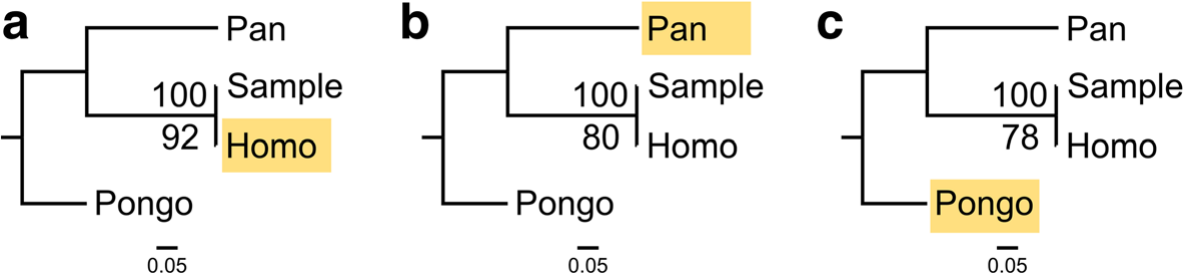
Phylogenetic trees for one human bone proteome dataset used in this study. **a** Phylogenetic tree resulting from the search against the *Homo* database. **b** Phylogenetic tree resulting from the search against the *Pan* database. **c** Phylogenetic tree resulting from the search against the *Pongo* database. Used database highlighted in yellow for each tree. The sample is placed identically due to the recovery of eight *Homo*-specific SAP variants in all three database searches. Upper nodal value: RAxML (0-100). Lower nodal value: PhyML (0-100)

The experiment conducted here indicates that unidentified mutable PSMs have a significant influence on the total number of proteins identified, but do not impede any subsequent phylogenetic analysis of the obtained data at an evolutionary distance of approximately 6-8 Ma (the split between *Homo* and *Pan*). However, at a larger evolutionary distance of approximately 16-18 Ma (the split between Ponginae and Homininae) a large proportion of phylogenetically informative positions is lost (up to 53%; Fig. 2d). The topology of *Pongo*, *Pan*, *Homo* and the proteomic sample resulting from all three searches is identical due to the consistent recovery of *Homo*-specific amino acid substitutions in all seven datasets (Fig. 6). Nevertheless, nodal support for the Sample + *Homo* node is decreased for PhyML in the searches against the *Pan* and *Pongo* database, compared to the *Homo* database. RAxML consistently recovers support for all nodes at 100%, however. This suggests that some phylogenetic methods are less suited when database and target species have relatively large suspected divergence times. The use of multiple methods of phylogenetic tree reconstruction should therefore be a requirement in ancient protein studies aiming to provide novel phylogenetic hypotheses for extinct species or populations.

The contribution of PSMs with two or more variable positions to sequence alignments is minimal as such PSMs will often go unidentified (≈75%), even in error-tolerant searches. This problem might not be particularly significant for proteins with relatively random, well-dispersed SAPs throughout their sequence. However, it has been demonstrated previously that SAPs for collagen type I, the dominant bone protein, are rather clustered and do not display a random distribution in either the *COL1α1* or *COL1α2* chains [1]. Similar patterns might be present for other collagenous and non-collagenous bone proteins as well. For such protein, the results of the experiment conducted here indicate that current error-tolerant algorithms are biased towards the identification of relatively conserved sequence regions. This is of significance, as recently acquired divergence dates for the lineages leading to *Macrauchenia* (an extinct South American Laurasiatherian mammal related to Perissodactyla) and *Equus* differ significantly when comparing estimates based on ancient protein sequences (≈74-89 Ma; [1, 3]) and ancient mitochondrial DNA sequences (≈56-78 Ma; [46]). This would be in line with the observations made in the current study. So, especially for divergence dating based on ancient proteins care must be taken as cross-species proteomic effects might compound age estimates due to non-random substitution patterns along protein sequences.

## Conclusions

The bioinformatic experiment presented here provides insights into the cross-species proteomic effects of standard and error-tolerant searches at increasing evolutionary distances. Incorrectly mutated peptide-spectrum matches are completely removed from the datasets using simple filtering criteria, providing experimental support for the use of error-tolerant search algorithms to identify novel hominin protein sequences in the future. Nevertheless, results suggest that there is an increasing loss of peptide and protein identifications at larger evolutionary distances, indicating that cross-species proteomic effects are not overcome completely by error-tolerant algorithms. The loss of peptide and protein identifications has significant effects on qualitative and quantitative proteome comparisons, especially for small proteomes such as those recovered in ancient (hominin) samples. Both peptide and protein identifications are biased towards the recovery of conserved amino acid sequences. As a result, divergence time estimates based on ancient protein datasets can be significantly overestimating true divergence times. This identification bias can be (partly) overcome by focusing on the generation of short peptides during protein extraction and digestion in future palaeoproteomics experiments, instead of focusing on longer peptides that would provide larger protein sequence coverage. Nevertheless, cross-species proteomic effects might still be prevalent when analyzing fast-evolving human proteins, for example those involved in immune responses, in the analysis of non-human bacterial proteins, or when focusing on proteins positively or negatively selected during animal and plant domestication. Cross-species proteomic effects are minimized between moderately divergent proteomes, as indicated by almost complete recovery of hominin SAPs in the search against the chimpanzee proteome (≈90%). This provides an experimental basis for the future phylogenetic analysis of ancient hominin protein sequences, including the identification of novel hominin single amino acid polymorphisms (SAPs) not present among currently available present-day human genomes or ancient modern human, Neanderthal, or Denisovan genomes.

## Additional files

Additional file 1: FASTA file containing possible contaminant proteins appended to each reference proteome. This list derives from the common Repository of Adventitious Proteins and contaminant proteins listed in [43]. (FASTA 39 kb)

Additional file 2: Additional parameters of unidentified, mutable PSMs. (a) Peptide isoelectric point (pI). (b) Peptide charge. (c) Differences in peptide mass. Searches against the *Pan* database are on the left, those to the *Pongo* database on the right. Point colour indicates the number of sequence differences between the *Homo* and *Pan*/*Pongo* sequence, respectively. Dashed lines indicate where isoelectric point, charge or peptide sequence mass is identical for homologous sequences. Peptide isoelectric point and charge are calculated at pH = 7. (PNG 586 kb)

## Abbreviations

*AHSG*: Alpha-2-HS-glycoprotein
*BGLAP*: Osteocalcin
*BGN*: Biglycan
*CHAD*: Chondroadherin
COL1: Collagen type I
*COL10α1*: Collagen type 10 alpha-1
*COL1α1*: Collagen type I alpha-1
*COL1α2*: Collagen type I alpha-2
FDR: False discovery rates
*FGA*: Fibrinogen alpha chain
LC-MS/MS: Liquid chromatography-tandem mass spectrometry
Ma: Million years
*OMD*: Osteomodulin
ppm: Parts per million
PSMs: Peptide spectrum matches
PTM: Post-translational modification
SAP: Single amino acid polymorphism
SNP: Single nucleotide polymorphism
