# Supplementary figures and images for: Elucidation of cross-species proteomic effects in human and hominin bone proteome identification through a bioinformatics experiment
Source: BMC Evol Biol. 2018 Feb 20;18:23. doi: 10.1186/s12862-018-1141-1 (PMC5819086; doi:10.1186/s12862-018-1141-1)

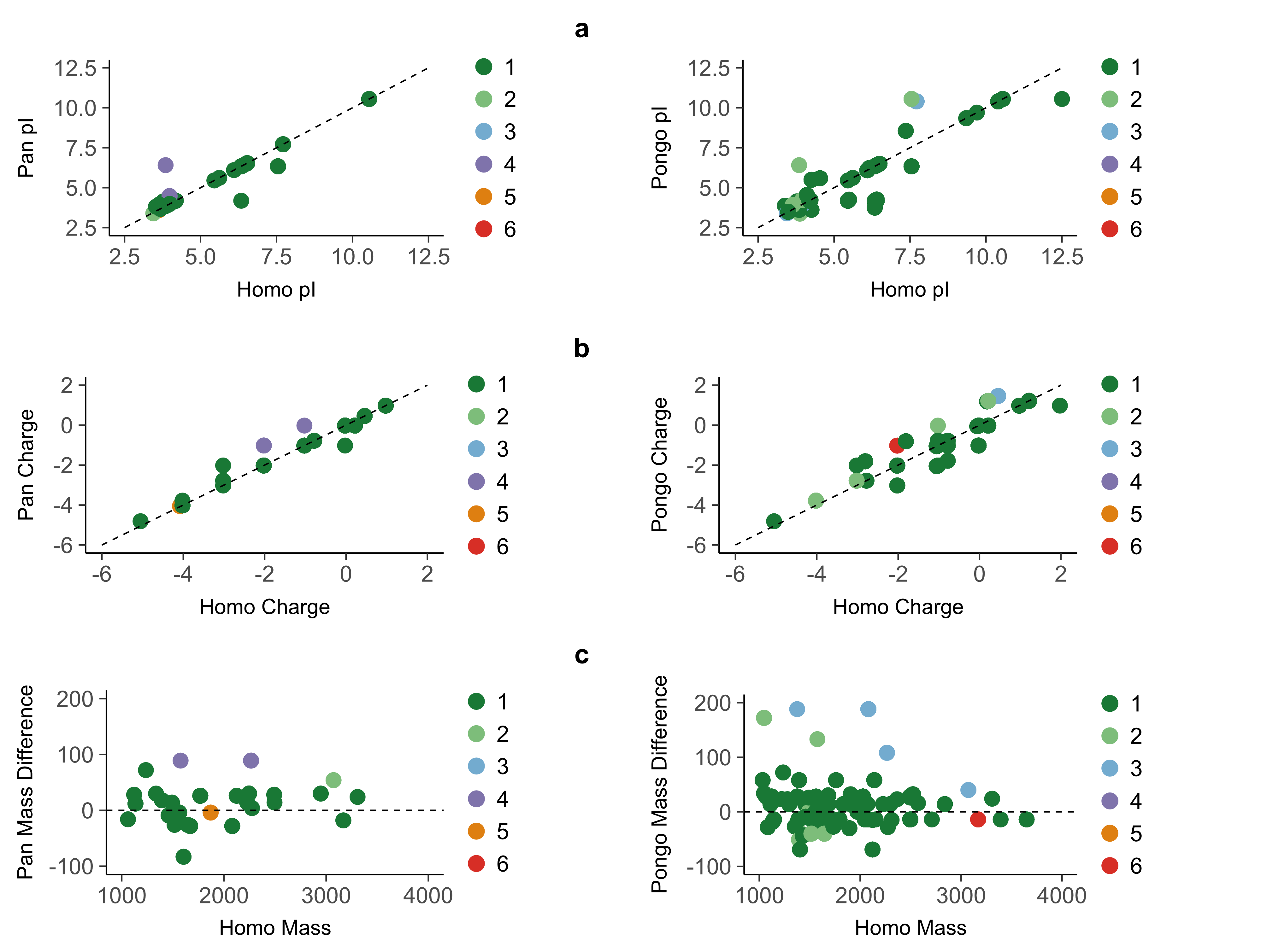

Supplement: Supplementary file 2 — Additional parameters of unidentified, mutable PSMs. (a) Peptide isoelectric point (pI). (b) Peptide charge. (c) Differences in peptide mass. Searches against the Pan database are on the left, those to the Pongo database on the right. Point colour indicates the number of sequence differences between the Homo and Pan/Pongo sequence, respectively. Dashed lines indicate where isoelectric point, charge or peptide sequence mass is identical for homologous sequences. Peptide isoelectric point and charge are calculated at pH = 7. (PNG 586 kb) [file 12862_2018_1141_MOESM2_ESM.png]
